# Klimawandel – ein Gesundheitsthema (auch) für Familien und ihre jungen Kinder?

**DOI:** 10.1007/s00103-024-03977-4

**Published:** 2024-11-21

**Authors:** Julia Schoierer, Hannah Lehmann, Johanna Köster-Lange, Jonas Gerke

**Affiliations:** 1 Institut und Poliklinik für Arbeits‑, Sozial- und Umweltmedizin, Klinikum der LMU München, Ziemssenstraße 5, 80336 München, Deutschland; 2 Ecolo – Agentur für Ökologie und Kommunikation, Bremen, Deutschland; 3 Deutsche Allianz Klimawandel und Gesundheit – KLUG e. V., Berlin, Deutschland

**Keywords:** Klimakompetenz, Hitzeschutz, Mentale Gesundheit, Klimaanpassung, Lebenswelten, Climate competence, Heat protection, Mental health, Climate adaptation, Personal living context

## Abstract

Nur auf einem gesunden Planeten können Kinder gesund aufwachsen. Der Klimawandel bedroht ihre Gesundheit durch steigende Temperaturen und extreme Wetterereignisse. In Deutschland sind die Durchschnittstemperaturen gestiegen, was zu häufigeren und intensiveren Hitzewellen führt. Diese Entwicklung gefährdet besonders Kinder, deren Thermoregulation noch nicht ausgereift ist. UV-Exposition erhöht das Risiko für Hautkrebs und Augenschäden. Wetterextreme beeinträchtigen zudem die mentale Gesundheit von Kindern.

Betreuungs‑, Bildungs- und Freizeiteinrichtungen spielen eine Schlüsselrolle im Umgang mit den psychischen und physischen Belastungen des Klimawandels. Widerstandsfähige Systeme und Einrichtungen können Stabilität und Resilienz fördern und präventiv wirken. Die Anpassung der Lebenswelten von Kindern an den Klimawandel ist jedoch noch unzureichend. Es ist essenziell, durch praxisnahe Informationsmaterialien die Klimakompetenz der Lebensweltakteurinnen und -akteure zu stärken, damit diese nicht nur sensibilisiert werden, sondern aktiv Schutzmaßnahmen etablieren können. Auch Krankenkassen können durch Beratung und Unterstützung klimafreundlicher und gesundheitsförderlicher Aktivitäten eine wichtige Rolle in der Prävention spielen.

Die Gesundheitsförderung in Lebenswelten mit Kindern birgt großes Potenzial, klimaschützendes und klimaangepasstes Verhalten zu fördern und langfristig bei Kindern und Familien zu etablieren. Um hier die notwendigen Veränderungen herbeizuführen, bedarf es der sektorenübergreifenden Zusammenarbeit zwischen Einrichtungen, Trägern, Behörden, Krankenkassen und -versicherungen und der Zivilgesellschaft sowie zwischen Bund, Ländern und Kommunen.

## Abstract (en)

Children can only grow up healthy on a healthy planet. Climate change threatens their health through rising temperatures and extreme weather events. In Germany, average temperatures have risen, leading to more frequent and intense heatwaves. This development particularly endangers children, whose thermoregulation is not yet fully developed. UV exposure increases the risk of skin cancer and eye damage. Extreme weather also negatively impacts children’s mental health.

Childcare, educational, and recreational facilities play a key role in managing the psychological and physical burdens of climate change. Resilient systems and facilities can promote stability and resilience and have a preventive effect. However, the adaptation of children’s personal living context to climate change is still inadequate. It is essential to enhance the climate competence of those responsible in these environments through practical informational materials that enable them to not only be aware but also to actively establish protective measures. Health insurance companies can also play a crucial role in prevention by advising and supporting climate-friendly and health-promoting activities.

Health promotion in environments with children has great potential to encourage climate-protective and climate-adaptive behavior and to establish it long term among children and families. Bringing about the necessary changes requires cross-sector collaboration between institutions, providers, authorities, health insurance companies, and civil society as well as between federal, state, and local governments.

## Hintergrund

Nur auf einem gesunden Planeten können Kinder gesund groß werden, denn wir Menschen stehen in einer ständigen Wechselwirkung mit unserer Umwelt.

Zerstörungen der Natur sowie der ungebremste Ausstoß von Treibhausgasen haben dazu geführt, dass sich unser Planet in den letzten Jahrzehnten massiv verändert hat. Die Temperaturen steigen immer weiter an – mit weitreichenden Folgen auch für unsere Gesundheit: häufigere, intensivere und länger andauernde Hitzewellen mit sogenannten Tropennächten, sich ändernde Vegetationszeiten mit Auswirkungen auf das Pollenfluggeschehen, häufigere Infektionskrankheiten durch Mücken oder Zecken, eine hohe Konzentration von Luftschadstoffen und Wetterextreme mit Unwettern und erhöhten Unfallgefahren. Die Erhöhung der globalen Mitteltemperaturen nähert sich deutlich den 1,5 °C an und in Deutschland verzeichnen wir mittlerweile sogar eine Temperaturerhöhung von 2,3 °C, betrachtet man die 10-Jahres-Abschnitte [1]. In Deutschland gilt die Hitze derzeit als gefährlichste Gesundheitsfolge für die Bevölkerung. Sie beeinträchtigt unsere Lebensqualität, unser Wohlempfinden und unsere Leistungsfähigkeit und kann zu schwerwiegenden Hitzeerkrankungen bis hin zum hitzebedingten Tod führen. Hitze betrifft die gesamte Bevölkerung, manche Personengruppen sind jedoch aufgrund des Zusammentreffens von Risikofaktoren besonders gefährdet, hitzebedingte Erkrankungen zu erleiden oder an der Hitze zu versterben.

Insgesamt sind Kinder, besonders Neugeborene, empfindlich gegenüber Klimafolgen. Ihr Körper befindet sich noch in der Entwicklung, sie haben eine höhere Stoffwechselrate und sie sind auf Unterstützung angewiesen. Zudem werden sie noch ihr ganzes Leben mit den Folgen des Klimawandels konfrontiert sein [2].

Vor welchen Herausforderungen stehen Familien mit Kindern derzeit und in Zukunft? Welche Unterstützungen brauchen sie und wer sind hier die relevanten Akteurinnen und Akteure?

Im Folgenden legen die Autorinnen und der Autor dar, welche gesundheitlichen Risiken sich für Kinder aus dem Klimawandel ergeben, wie sich Strukturen und Abläufe in den Lebenswelten von Kindern gesundheitsförderlich und klimaresilient gestalten lassen und wie Verantwortliche und Multiplikatoren in diesem Prozess gemeinsam agieren können.

## Klimawandel und Kindergesundheit

### Hitzebelastung

2023 war wieder ein Rekordjahr in Temperaturen und mit inzwischen 2,3 °C Erwärmung liegt Deutschland unter Betrachtung von Temperaturanomalien in 10-Jahres-Perioden deutlich über den globalen Mitteltemperaturen [1]. Höchsttemperaturen bis zu 40 °C sind auch in Deutschland keine Seltenheit mehr. Es wird zunehmend zu Hitzeereignissen kommen, die länger andauern, intensiver (also mit höheren Temperaturen) ausfallen und auch früher im Jahr stattfinden. In den letzten Jahren konnte der Deutsche Wetterdienst (DWD) die ersten Hitzewellen bereits im Mai verzeichnen – zu einem Zeitpunkt, an dem die Menschen auf solche Hitzeereignisse noch gar nicht vorbereitet sind und auch der Körper noch nicht akklimatisiert ist. Dabei gibt es regionale und lokale Unterschiede. Die Regionen um den Mittelrheingraben und in Brandenburg sind eher betroffen, Städte verzeichnen höhere Temperaturen als ländliche Regionen. Aufgrund von Versiegelungen, fehlenden Frischluftschneisen, fehlendem Grün und Blau (z. B. Grünflächen, Bäume oder begrünte Fassaden sowie Stadtbäche, Teiche oder Brunnen) und der Abstrahlwärme von Häuserflächen können nächtliche Temperaturen in städtischen Wärmeinseln um 10 °C höher sein als im Umland.

Hitze wird derzeit als die größte klimawandelbedingte Gesundheitsgefahr in Deutschland eingeordnet [3]. Sie betrifft alle Menschen und schränkt nicht nur unser Wohlbefinden ein, sie kann auch zu lebensbedrohlichen Erkrankungen führen bis hin zum Tod. Aber nicht alle Menschen sind gleichermaßen von Hitze betroffen. Die Betroffenheit ist sehr individuell und beruht auf physischen sowie soziodemografischen Faktoren und den Anpassungsmöglichkeiten bis hin zur Exposition.

Bisher standen vor allem die Menschen im höheren Lebensalter im Fokus. Zweifelsohne hat diese Personengruppe durch Hitze ein besonders hohes Risiko, schwer zu erkranken oder sogar zu versterben. Denn im hohen Alter treffen hitzerelevante Risikofaktoren oftmals zusammen, z. B. verringerte Thermoregulation, chronische Erkrankungen, Medikamenteneinnahme, Einschränkungen in der Selbstversorgung und ein hoher Unterstützungsbedarf. Verstärkt wird das Risiko noch durch Einsamkeit, Isolation, fehlende Kontakte zu Angehörigen oder in der Nachbarschaft sowie eine sich stark aufheizende Wohnumgebung oder das Leben in einer städtischen Wärmeinsel.

Die Studienlage zeigt aber immer stärker die Vielzahl gefährdeter Personengruppen sowie die Heterogenität innerhalb der Gruppen, je nachdem, welche Risiko-, aber auch Resilienzfaktoren zusammentreffen. Dabei spielen die Lebenswelten, in denen sich die Menschen aufhalten, eine entscheidende Rolle. Diese kann protektiv, aber auch belastend sein, je nachdem, welche Hitzebelastung dort herrscht und welche Hitzekompetenz die Akteurinnen und Akteure der jeweiligen Lebenswelten aufweisen.

Neugeborene, Säuglinge und Kinder sind aufgrund ihrer Physiologie bereits stärker durch Hitze gefährdet (s. Infobox). Ihre Thermoregulation ist noch nicht voll ausgereift, sie schwitzen weniger und aufgrund des Verhältnisses von Körperoberfläche zu Körpervolumen muss ihr Körper mehr arbeiten, um den Kühlmechanismus umzusetzen (Blut zur Wärmeabgabe an die Hautoberfläche transportieren). Kinder bewegen sich mehr und haben eine höhere Stoffwechselrate. Oftmals sind sie so in ihr Spiel vertieft, dass sie nicht daran denken zu trinken und sie empfinden auch weniger Durst. Kinder sind also je nach Lebensalter auf Unterstützung angewiesen, können die Gefahr von Hitze noch nicht adäquat einschätzen und insbesondere im Säuglingsalter dieser auch nicht selbst ausweichen. Zudem befinden sie sich oftmals ganztägig in Betreuungseinrichtungen, die wenig Flexibilität zulassen. Verfügt das pädagogische Personal über eine geringe Hitzekompetenz oder sind keine Hitzeschutzmaßnahmen in der Einrichtung etabliert, so können Kitas, Schulen, Sportvereine oder Freizeitangebote zu einem Risikofaktor bei Hitze werden, aber genauso auch zu einem Resilienzfaktor, sobald die Kinder in der Einrichtung und ihrer Freizeit vor Hitze geschützt werden können. So sieht es auch in ihrem Zuhause aus: Sind die Familien in der Lage, sich vor Hitze zu schützen, sowohl durch ihr Verhalten als auch durch die Verhältnisse, in denen sie leben, hat dies eine direkte Auswirkung auf die Hitzebelastungen der Kinder.

#### Infobox

Kinder als Risikogruppe für Hitze- und UV-Belastung.

Je jünger das Kind, desto empfindlicher ist es gegenüber Hitze- und UV-Belastung:

- Regulation der Körpertemperatur ist nicht ausgereift
- Die Kinderhaut ist sehr empfindlich
- Kinder halten sich mehr im Freien auf
- Es besteht kein Risikobewusstsein
- Es besteht hoher Unterstützungsbedarf
- Die Kinder befinden sich in Lebenswelten, die schützend aber auch belastend wirken können, je nach
  - Hitze- und UV-Exposition
  - Umsetzung entsprechender Schutzmaßnahmen
  - (Hitze- und UV-)Kompetenz der Lebensweltakteurinnen und -akteure
- Kinder haben nur begrenzte Entscheidungsmöglichkeiten

### UV-Belastung

Mit dem Klimawandel geht auch eine erhöhte UV-Exposition einher. Generell steigt die Anzahl der Sonnenscheinstunden aufgrund des Klimawandels an und somit auch die UV-Belastung. Seit Messbeginn 1951 hat hierzulande die Sonnenscheindauer im Mittel um gut 10 % zugenommen [1]. Aufgrund des sonnigeren und warmen/heißen Wetters verbringen die Menschen mehr Zeit im Freien und setzen ihre Haut mehr UV-Strahlung aus.

Die kindliche Haut ist gegenüber UV-Strahlung besonders empfindlich. Sonnenbrände in der Kindheit erhöhen das Risiko für den schwarzen Hautkrebs um das 2‑ bis 3‑Fache. Auch die Augen sind gefährdet, denn die UV-Belastung erhöht das Risiko für einen grauen Star (Linsentrübung). Trotz intensiver Präventionsarbeit, die seit Jahren geleistet wird, hat sich seit dem Jahr 2000 die Zahl der Hautkrebserkrankungen mehr als verdoppelt [4]. Und wie beim Hitzeschutz gilt die Lebenswelt der Kinder als entscheidender Faktor der UV-Belastung.

### Mentale Gesundheit

Insbesondere die durch den Klimawandel zunehmenden Wetterextreme wie Stürme, Unwetter, Hitze, Dürren und Starkniederschläge können neben den direkten körperlichen Auswirkungen auch mentale Belastungen mit sich bringen [5]. Dabei sind sowohl das unmittelbare Erleben des Ereignisses als auch die weitreichende Zerstörung der vertrauten Umgebung und des sozialen Netzwerks sowie der Verlust von Angehörigen von Bedeutung [6]. Kinder und Jugendliche sind besonders anfällig für die psychischen Folgen solcher Ereignisse, da sie im Vergleich zu Erwachsenen über weniger ausgeprägte Bewältigungsstrategien verfügen [7]. Als Reaktion können posttraumatische Stresssymptome bzw. Belastungsstörungen sowie gesteigerte Anspannung und aggressives Verhalten, Schlafstörungen, Lern- und Konzentrationsschwierigkeiten, Panikattacken, Angststörungen, Depressionen und Substanzmissbrauch auftreten [8–10]. Der Klimawandel und seine Folgen können aber auch ohne direktes Erleben eines Extremereignisses zu indirekten psychischen Auswirkungen führen. Insbesondere bei jungen Menschen gewinnen Klimaemotionen und Klimaangst an Bedeutung, die negative Emotionen und Symptome wie Panikattacken, Appetitlosigkeit, zwanghaftes Denken und Schlaflosigkeit nach sich ziehen können [11, 12].

Es hat sich gezeigt, dass Betreuungs‑, Bildungs- und Freizeiteinrichtungen vor allem im Umgang mit den psychischen Belastungen, die Kinder und Jugendliche aufgrund der Auswirkungen des Klimawandels erfahren, ein wichtiger Anker sind [13]. Zum einen können widerstandsfähige Systeme und Einrichtungen in Krisenzeiten Stabilität und Kontinuität bieten. Zum anderen können Programme zur Förderung der Resilienz, die Vermittlung von Bewältigungskompetenzen und die Erfahrung von Selbstwirksamkeit innerhalb dieser Einrichtungen präventiv wirken [14].

## Risiko oder Schutz durch die Lebenswelten

### Anpassung der Strukturen und Abläufe an die Folgen des Klimawandels

In Deutschland ist der Schutz vor den Folgen des Klimawandels in Kitas und Schulen noch nicht ausreichend etabliert, viele Einrichtungen und Kommunen sind auf diese Herausforderung noch nicht vorbereitet [15–17].

Dies bestätigte auch eine Studie aus dem Jahr 2023, die unter pädagogischem Personal in Kindertageseinrichtungen in München durchgeführt wurde. Hierbei wurden 181 Personen dazu befragt, wie sie das Risiko klimawandelbedingter Gesundheitsgefahren im Hinblick auf die Kindergesundheit und die eigene Gesundheit am Arbeitsplatz heute und in den nächsten 10 Jahren einschätzen und ob bzw. welche Maßnahmen zur (gesundheitsbezogenen) Anpassung an den Klimawandel in den Kindertagesstätten umgesetzt werden. Es zeigte sich, dass trotz der starken Wahrnehmung von klimawandelbedingten Gesundheitsrisiken die Umsetzung entsprechender Schutz- und Anpassungsmaßnahmen in der Mehrzahl der befragten Kitas bisher unzureichend ist. Auch in der pädagogischen Arbeit mit den Kindern spielte das Thema Klimawandel eine eher untergeordnete Rolle. Die Informiertheit des pädagogischen Personals über das Thema Klimawandel und Gesundheit erwies sich jedoch als ein entscheidender Einflussfaktor bei der Umsetzung von Maßnahmen [17].

Schwierig ist oftmals die Umsetzung von Maßnahmen, die bauliche oder technische Gegebenheiten betreffen. Gebäude und Außenbereiche sind nicht ausreichend an die neuen Bedingungen angepasst und es mangelt an Begrünung sowie Beschattung [16, 18]. Die Begrünung und (mobile) Verschattung von Spielplätzen und Außenflächen in Kitas und Schulen oder das Aufstellen von Trinkbrunnen sind jedoch gute Beispiele dafür, dass bauliche Maßnahmen einen wesentlichen Beitrag zur Anpassung an die Klimawandelfolgen leisten, insbesondere an die erhöhte Hitzebelastung.

Neben langfristigen Anpassungsmaßnahmen ist auch ein akuter Schutz vor Klimawandelfolgen erforderlich. In Kitas werden als Schutz vor der zunehmenden UV-Strahlung bereits einzelne Vorsorge- oder Schutzmaßnahmen praktiziert, wie z. B. regelmäßiges Eincremen mit Sonnencreme, das Tragen von Kopfbedeckungen und der Aufenthalt im Schatten [16, 19]. Sinnvoll wäre ein systematisches Vorgehen, das strukturiert sowohl präventive Maßnahmen als auch Sofortmaßnahmen umfasst. Während es für Kitas teilweise bereits einrichtungsspezifische Handlungsempfehlungen gibt, fehlen für Schulen, Freizeit- und Sportvereine und andere Lebenswelten entsprechende Empfehlungen weitgehend [17, 20, 21].

In den letzten Jahren wurden in Bezug auf die Gefahr durch Hitze Hitzeschutzpläne oder Hitzeaktionspläne entwickelt, um mithilfe verhaltens- und verhältnispräventiver Maßnahmen hitze- und UV-bedingten Erkrankungen vorzubeugen [22, 23]. Diese Entwicklung findet bislang hauptsächlich auf Ebene der Kommunen statt. Einrichtungsspezifische Maßnahmenpläne für Kitas, Schulen und Sportvereine können jedoch effektive Mittel sein, um die Risiken durch hohe Hitzebelastung zu mindern. Dazu gehören langfristige Anpassungsmaßnahmen, wie z. B. Beschattung oder Begrünung von Außenfassaden und Dächern oder die Reduzierung von Flächenversiegelung, ebenso wie kurz- und mittelfristige Verhaltensanpassungen und festgeschriebene Abläufe bei bestimmten Warnstufen. Diese Prozesse müssen auch durch bessere personelle und finanzielle Ressourcen begleitet werden, um auf die Herausforderungen in der Praxis einzugehen.

Außerdem ist die Einbindung von Akteurinnen und Akteuren innerhalb der jeweiligen Lebenswelt, hier als Lebensweltakteurinnen und -akteure bezeichnet, in die Entwicklung und Umsetzung von Klimaanpassungsmaßnahmen von zentraler Bedeutung, wie z. B. des pädagogischen Fachpersonals. Maßnahmenpläne müssen aufzeigen, wie die klimatischen Veränderungen vor Ort gemeistert werden können, und sie müssen beachten, welche Möglichkeiten und Ressourcen in der Einrichtung vorhanden sind. Partizipative Prozesse können dabei unterstützen, alle Mitarbeitenden zu befähigen, selbst Lösungen zu entwickeln [19].

Für die praxisnahe Umsetzung ist ein Überblick jeweils darüber notwendig, ob und wo Ansätze bestehen bzw. was bereits unternommen wird und wo die Herausforderungen bei der Umsetzung dieser Ansätze liegen. Der Austausch und die Vernetzung zwischen den Akteurinnen und Akteuren spielen dabei eine wichtige Rolle, z. B. in Form eines Austauschs von Good-Practice-Beispielen.

### Hitzekompetenz der Verantwortlichen erhöhen

Zusätzlich muss die Hitzekompetenz der verantwortlichen Personen gestärkt werden. Lebensweltakteurinnen und -akteure müssen in die Lage versetzt werden, auf die gesundheitlichen Gefahren durch die Klimaveränderungen zu reagieren und ihre Verhaltensweisen anzupassen. Informative und praxisnahe Informationsmaterialien wie Leitfäden, Musterpläne und Schulungen können hierbei unterstützend wirken [17]. Gut aufbereitete Materialien und umfassende Informationen werden vom Zentrum Klima Anpassung, dem Webangebot www.hitzeservice.de, der Deutschen Allianz Klimawandel und Gesundheit (KLUG), verschiedenen Landesvereinigungen für Gesundheitsförderung sowie der Bundeszentrale für gesundheitliche Aufklärung (BZgA) angeboten. Informationsmaterial kann aber nur eine Unterstützung bieten, denn entscheidend für die Erhöhung der Hitzekompetenz ist die direkte Ansprache.

Der Zusammenhang von Klimawandel und Gesundheit beschränkt sich nicht auf die entstehenden Gesundheitsrisiken. Neben dem Schutz vor den Folgen des Klimawandels und der Vorbereitung auf Extremwetterereignisse ist es notwendig, die Bedingungen innerhalb der sich verändernden Lebenswelten gesundheitsförderlich zu gestalten. Lebensweltbezogene Aktivitäten zur Gesundheitsförderung und Prävention sind ebenfalls entscheidend, um den mit dem Klimawandel einhergehenden gesundheitlichen Risiken und letztendlich auch dem Klimawandel selbst entgegenzuwirken. Auch hier sind die Sensibilisierung und Bewusstseinsbildung der Lebensweltakteurinnen und -akteure zentral.

In ihrer Rolle als Vorbilder für Kinder können Lebensweltakteurinnen und -akteure als Multiplikatorinnen und Multiplikatoren wirken und die Sensibilisierung von Kindern und dadurch auch von Familien durch zielgruppenspezifische Aufklärung und Kompetenzentwicklung vorantreiben. Der positive Effekt von generationsübergreifendem Lernen von Kindern zu Eltern ist bereits wissenschaftlich belegt. Es konnte gezeigt werden, dass Kinder mithilfe ihres erlernten Wissens Erwachsene und insbesondere ihre Eltern dazu motivieren können, sich stärker mit dem Thema Klimawandel und dessen Folgen sowie den Handlungsmöglichkeiten zu beschäftigen [24, 25]. Eltern sollten ebenfalls in ihrer besonderen Vorbildfunktion für klimaschützendes und -angepasstes Verhalten unterstützt werden.

Neben der Wissensvermittlung sowie der Sensibilisierung und Bewusstseinsbildung bei Lebensweltakteurinnen und -akteuren ist es wichtig, praktische Ansätze und Umsetzungsbeispiele zu vermitteln und praktische Erfahrungen zu klimaschützenden und -anpassenden Maßnahmen zu ermöglichen. Dies kann durch gemeinsame Projekte und Aktionen mit Lebensweltakteurinnen und -akteuren, Eltern und Kindern erreicht werden. Denn neben der Risikowahrnehmung und der direkten Betroffenheit spielt die Überzeugung von der Wirksamkeit und Umsetzbarkeit der Maßnahmen eine große Rolle für die Motivation, Vorsorge gegen die Folgen des Klimawandels zu treffen [26].

Eine zentrale Bedeutung kommt den Krankenkassen als Multiplikatoren zu, indem sie Klimaresilienz und Klimakompetenz in der Gesellschaft fördern und einen kompetenten Umgang mit den Folgen des Klimawandels vermitteln. Sie können Einrichtungen bei der Anpassung unterstützen, indem sie klimaschützende und -anpassende Elemente in gesundheitsförderliche und präventive Aktivitäten integrieren, z. B. durch Wissensvermittlung zu spezifischen Themen wie Hitze, durch Anpassung von bestehenden Angeboten oder durch Bereitstellung von Strukturen und Netzwerken zum Klimaschutz und zur Klimaanpassung [27].

Durch den im Dezember 2022 neu gefassten „Leitfaden Prävention“ [28] haben die Krankenkassen neue Möglichkeiten erhalten, Verantwortliche in Lebenswelten bei gesundheitlich relevanten Aspekten des Klimaschutzes und der Klimaanpassung, insbesondere beim Hitzeschutz, zu unterstützen. Durch die Änderungen können Krankenkassen bei der gesundheitsförderlichen Ausrichtung von Kitas und Schulen mitwirken, gesunde und klimafreundliche Verpflegung und Mobilität fördern sowie Kommunen bei der Prävention klimawandelbedingter Gesundheitsrisiken unterstützen. Ein besonderer Fokus liegt hierbei auf der Aus- und Weiterbildung der Lebensweltakteurinnen und -akteure.

Im Jahr 2023 haben bereits mehrere Krankenkassen ihr Profil neu ausgerichtet, um Lebenswelten beim Klimaschutz und der Klimaanpassung zu unterstützen und den gesundheitlichen Auswirkungen des Klimawandels entgegenzuwirken. Eine zentrale Rolle spielen dabei Mitarbeitende im Bereich Gesundheitsförderung und Prävention, die die neuen Themen in ihr Beratungsangebot integrieren.

### Planetary Health, Co-Benefits, Klimaanpassung als Gemeinschaftsaufgabe

Maßnahmen des Klimaschutzes und der Klimaanpassung sind eng miteinander verknüpft. Die Prävention klimawandelbedingter Gesundheitsgefahren ist bis zu einem gewissen Punkt durch verhaltens- oder verhältnispräventive Anpassungen möglich, doch letztendlich muss sie auch den Schutz der Atmosphäre vor weiterer Erwärmung umfassen.

Das Konzept „Planetary Health“ hilft dabei, die Zusammenhänge zwischen der Gesundheit von Kindern und Familien und Umweltveränderungen besser zu verstehen. Eine gesunde natürliche Umwelt wird als Grundlage eines gesunden Lebens verstanden. Maßnahmen für Klimaschutz und -anpassung können Zusatznutzen für die Gesundheit schaffen, sogenannte Co-Benefits. Ein Beispiel hierfür ist die Ernährung als ein wichtiger Schlüssel für gesunde Familien und ein gesundes Klima. Kitas und Schulen können durch die Förderung klimafreundlicher und gesundheitsförderlicher Gemeinschaftsverpflegung einen wichtigen Beitrag zum Klimaschutz leisten und ernährungsbedingte Gesundheitsrisiken verringern. Zur Orientierung gibt es Empfehlungen der Deutschen Gesellschaft für Ernährung e. V. für Kitas, Schulen und andere Gemeinschaftseinrichtungen sowie die Ernährungsempfehlung „Planetary Health Diet“ der EAT-Lancet Commission [29]. Auch bei den Themen Bewegung und Mobilität gibt es Co-Benefits. Durch die Schaffung von sicheren, klimaangepassten Wegen (z. B. Fuß- oder Radwege zur Schule und Kita) und klimaangepassten Freiflächen (z. B. Spiel- und Sportflächen, öffentliche Plätze und Parks) lassen sich Kinder an eine aktive Mobilität und Bewegung heranführen. Langfristig wirkt sich dies positiv auf die Gesundheit und die körperliche sowie motorische Entwicklung aus und reduziert gleichzeitig den CO_2_-Ausstoß sowie die Schadstoff- und Lärmbelastung [30–33].

Klimaschutz und Klimaanpassung innerhalb der Lebenswelten bieten in diesem Kontext eine ausgezeichnete Gelegenheit, Kindern Selbstwirksamkeit zu vermitteln und sie aktiv einzubinden. Kinder zeigen oft große Begeisterung und Neugierde für neue Kompetenzen und Aufgaben. Gesundheitsförderung in Lebenswelten mit Kindern birgt großes Potenzial, klimaschützendes und klimaangepasstes Verhalten zu fördern und langfristig bei Kindern und Familien zu etablieren. Familien – besonders in der frühen Lebensphase der Kinder – haben ein großes Interesse an der gesunden Entwicklung ihres Kindes.

Eltern spielen eine wichtige Rolle dabei, Kinder vor den gesundheitlichen Folgen des Klimawandels zu schützen. Die Steigerung ihrer Klimakompetenz sollte ein weiterer Fokus sein. Es sollte Angebote geben, die Eltern auch direkt zu den gesundheitlichen Folgen des Klimawandels schulen, um die gesundheitlichen Gefahren ihrer Kinder zu verstehen und zu deren Schutz beizutragen.

Diese Aufgabe ist jedoch nur gemeinschaftlich zu schaffen. Es bedarf einer sektorenübergreifenden Zusammenarbeit zwischen den Einrichtungen, Trägern, Behörden, Krankenkassen und der Zivilgesellschaft sowie zwischen Bund, Ländern und Kommunen. Es müssen neue Bündnisse geschaffen werden, um konkrete Veränderungen herbeizuführen. Das Netzwerk der Frühen Hilfen könnte hierfür ein gutes Vorbild sein, welches sich u. a. durch eine interprofessionelle Zusammenarbeit von Fachkräften unterschiedlicher Lebenswelten auszeichnet. Aber auch weitere bestehende Strukturen und Zugangswege zu Familien, wie z. B. Geburtsvorbereitungskurse, Hebammenbetreuung, Früherkennungsuntersuchungen oder pädagogische Angebote, sollten genutzt werden.

## Fazit

Gesundheitliche Klimaanpassung nur auf den Aspekt Sensibilisierung zu stützen ist zu kurz gegriffen. Es müssen immer auch die jeweilige Verwundbarkeit und Exposition betrachtet werden. Wenn Risikofaktoren in den Lebenswelten, Expositionen im öffentlichen Raum und der Wohnumgebung identifiziert sind, können diese reduziert werden und die Gesundheit von Kindern im Sinne des Präventionsgedankens geschützt werden. Gesundheitsförderliche und klimaresiliente Lebenswelten bieten die große Chance, die Kompetenzen durch die Weitergabe des Wissens und der Fähigkeiten im Umgang mit den Auswirkungen des Klimawandels generationsübergreifend in die Familien zu tragen und damit zum dringend notwendigen gesamtgesellschaftlichen Transformationsprozess beizutragen.

Dabei ist das Verstehen dieser Aufgabe als sektorenübergreifende Zusammenarbeit entscheidend, in der die verschiedenen Akteursgruppen der jeweiligen Lebenswelten miteinander agieren. Betrachtet man die Lebenswelten von Kindern und deren Familien, so finden sich Akteurinnen und Akteure sowie Zugangswege:

- in der Gesundheitsversorgung durch pädiatrische Praxen, therapeutische Angebote, Apotheken, Krankenhäuser sowie durch Hebammen,
- über die Krankenkassen,
- über die Kommunen durch die Frühen Hilfen, Familienbüros oder auch das Gesundheitsamt,
- in pädagogischen Einrichtungen sowie im Sport- und Freizeitangebot.

Das Abstimmen von Aktivitäten sowie der Austausch untereinander sind von großer Bedeutung, um im Rahmen von gesundheitlicher Klimaanpassung dieselbe Botschaft zu vermitteln und die Klimakompetenz zu fördern. Wie in Tab. 1 dargestellt, müssen dabei keine neuen Strukturen geschaffen, sondern vorhandene Strukturen und Kommunikationskanäle mit Themen der gesundheitlichen Klimaanpassung bespielt werden.

**Tab. 1:** Zugangswege, Akteursgruppen und Kommunikationskanäle in der gesundheitlichen Klimaanpassung. (Adaptiert nach [34])

Zugangswege	Akteurinnen und Akteure	Kommunikationskanäle
Gesundheitsversorgung	Hebammen, pädiatrische Praxis, Krankenhaus, therapeutische Angebote, Apotheken	Hausbesuche, Kurse, Beratung, Vorsorgeuntersuchungen, Sprechstunden, Behandlungen, Kurse, Informationsmaterial im Wartezimmer
Krankenkassen	GKV/PKV, Geschäftsstellen, Mitgliederberatung, Kommunikationsabteilungen	Mitgliederzeitschriften, Anschreiben, Informationsmaterialien, Apps, Kursangebote, Hotlines
Kommunen	Frühe Hilfen, Familienbüros, Gesundheitsamt	Beratung, Hausbesuche, Veranstaltungen, Schuleingangsuntersuchungen, Informationsmaterial
Pädagogische Einrichtungen	Kitas, Schulen	Schulunterricht, Betreuungszeit, Elternabende, Anschreiben, Veranstaltungen, Informationsmaterial
Sport- und Freizeiteinrichtungen	Trainer, pädagogisches Personal, Betreuer	Training, Veranstaltungen, Kinderfreizeiten, Informationsmaterial

## References

[1] Deutscher Wetterdienst (2006) Deutschlandwetter im Jahr 2023. https://www.dwd.de/DE/presse/pressemitteilungen/DE/2023/20231229_deutschlandwetter_jahr2023_news.html. Zugegriffen: 19. Juli 2024

[2] Schoierer J, Lehmann H (2023) Klimawandel-Hitze-Kindergesundheit. Pflege Z 74:22–25. 10.1007/s41906-023-2040-7

[3] Kahlenborn W, Porst L, Voss M et al (2021) Climate impact and risk assessment 2021 for Germany. Umweltbundesamt (UBA)

[4] Baldermann C, Weiskopf D (2020) Behavioral and structural prevention of skin cancer: implementation and effectiveness. Hautarzt 71:572–579. 10.1007/s00105-020-04613-3 32494842 10.1007/s00105-020-04613-3

[5] Watts N, Amann M, Arnell N et al (2019) The 2019 report of the Lancet countdown on health and climate change: ensuring that the health of a child born today is not defined by a changing climate. Lancet 394(10211):1836–1878. 10.1016/S0140-6736(19)32596-6 31733928 10.1016/S0140-6736(19)32596-6 PMC7616843

[6] Burke SE, Sanson AV, Van Hoorn J (2018) The psychological effects of climate change on children. Curr Psychiatry Rep 20:1–8. 10.1007/s11920-018-0896-9 29637319 10.1007/s11920-018-0896-9

[7] Garcia DM, Sheehan MC (2016) Extreme weather-driven disasters and children’s health. Int J Health Serv 46(1):79–105. 10.1177/0020731415625254 26721564 10.1177/0020731415625254

[8] Fernandez A, Black J, Jones M et al (2015) Flooding and mental health: a systematic mapping review. PLoS ONE. 10.1371/journal.pone.0119929 25860572 10.1371/journal.pone.0119929 PMC4393088

[9] Mambrey V, Wermuth I, Boese-O’Reilly S (2019) Auswirkungen von Extremwetterereignissen auf die psychische Gesundheit von Kindern und Jugendlichen. Bundesgesundheitsblatt Gesundheitsforschung Gesundheitsschutz 62(5):599–604. 10.1007/s00103-019-02937-7 30976819 10.1007/s00103-019-02937-7

[10] Maclean JC, Popovici I, French MT (2016) Are natural disasters in early childhood associated with mental health and substance use disorders as an adult? Soc Sci Med 151:78–91. 10.1016/j.socscimed.2016.01.006 26789078 10.1016/j.socscimed.2016.01.006

[11] Stanley SK, Hogg TL, Leviston L et al (2021) From anger to action: differential impacts of eco-anxiety, eco-depression, and eco-anger on climate action and wellbeing. J Clim Chang Health 1:100003. 10.1016/j.joclim.2021.100003

[12] Usher K, Durkin J, Bhullar N (2019) Eco-anxiety: how thinking about climate change-related environmental decline is affecting our mental health. Int J Mental Health Nurs. 10.1111/inm.12673 10.1111/inm.12673 31724833

[13] Schürr A, Elbel J, Hieronimi A, Auer I, Coenen M, Böse-O’Reilly S (2023) Mental health in adolescents after experiencing a flood event in Bavaria, Germany—a qualitative interview study. Front Public Health 11:1210072. 10.3389/fpubh.2023.1210072 37744475 10.3389/fpubh.2023.1210072 PMC10511869

[14] Peter F, van Bronswijk K (2021) Die Klimakrise als Krise der psychischen Gesundheit für Kinder und Jugendliche. Pädiatr Allergol 03:58–63. 10.13109/9783666407710.159

[15] Malmquist A, Lundgren T, Hjerpe M, Glaas E, Turner E, Storbjörk S (2021) Vulnerability and adaptation to heat waves in preschools: experiences, impacts and responses by unit heads, educators and parents. Climate Risk Manag 31:100271. 10.1016/j.crm.2020.100271

[16] Lehmann H, Böse-O’Reilly S, Schoierer J, Garschagen M (2023) Climate change-related health hazards in daycare centers in Munich, Germany: risk perception and adaptation measures. Reg Environ Change 23(4):147. 10.1007/s10113-023-02136-w

[17] Heidenreich A, Deppermann LH, Thieken AH, Otto A (2024) Maßnahmen zur Hitze- und Starkregenvorsorge in Kitas und Pflegeeinrichtungen: Eine Evaluation von Risikowahrnehmung, Kommunikation und Informationsmaterialien. Bundesgesundheitsblatt Gesundheitsforschung Gesundheitsschutz. 10.1007/s00103-024-03876-8 38656348 10.1007/s00103-024-03876-8 PMC11166820

[18] ThINK – Thüringer Institut für Nachhaltigkeit und Klimaschutz GmbH (2017) Untersuchung der Wärmebelastung an kommunalen Kindertagesstätten und Grundschulen der Stadt Jena. https://sessionnet.jena.de/sessionnet/buergerinfo/getfile.php?id=79781&type=do&. Zugegriffen: 22. Juli 2024

[19] Geene R, Hartung E, Grapetin N et al (2023) Abschlussbericht Ansätze zur klimagesunden Prävention und Gesundheitsförderung in Lebenswelten (KliGes). Resource document. http://bigso.de/gallery/23-05-22%20KliGeS_Abschlussbericht_BIGSo2.pdf. Zugegriffen: 22. Juli 2024

[20] Schwartz C (2023) Philologenverband fordert Hitzeschutzplan für rheinland-pfälzische Schulen – Initiative des Bundesgesundheitsministers muss von Landesregierung aufgegriffen werden. https://www.philologenverband.de/diverses/texte/news/philologenverband-fordert-hitzeschutzplan-fuer-rheinland-pfaelzische-schulen-initiative-des-bundesge.html?tx_news_pi1%5Bcontroller%5D=News&tx_news_pi1%5Baction%5D=detail&cHash=79d2d3443e9d25130b78e71e2. Zugegriffen: 22. Juli 2024

[21] Deutsches Rotes Kreuz (2023) Klimaanpassung und Klimaschutz in DRK-Kindertageseinrichtungen. https://drk-wohlfahrt.de/fileadmin/DRK-Wohlfahrt.de/02-Themen/Kinder-Jugend-Familie/Kinder/Klima/DRK_Praxishandreichung_Klima-Kita_Interaktiv_20231025_DS__1_.pdf. Zugegriffen: 22. Juli 2024

[22] Straff W, Mücke HG (2017) Handlungsempfehlungen für die Erstellung von Hitzeaktionsplänen zum Schutz der menschlichen Gesundheit. Resource document. https://www.bmu.de/fileadmin/Daten_BMU/Download_PDF/Klimaschutz/hap_handlungsempfehlungen_bf.pdf. Zugegriffen: 22. Juli 2024 10.1007/s00103-017-2554-5 28492969

[23] Blättner B (2021) Arbeitshilfe zur Entwicklung und Implementierung eines Hitzeaktionsplans für Städte und Kommunen. https://www.hs-fulda.de/fileadmin/user_upload/FB_Pflege_und_Gesundheit/Forschung___Entwicklung/Arbeitshilfe_Hitzeaktionsplaene_in_Kommunen_2021.pdf. Zugegriffen: 22. Juli 2024

[24] Lawson DF, Stevenson KT, Peterson MN et al (2018) Intergenerational learning: are children key in spurring climate action? Glob Environ Change 53:204–208. 10.1016/j.gloenvcha.2018.10.002

[25] Lawson DF, Stevenson KT, Peterson MN et al (2019) Children can foster climate change concern among their parents. Nat Clim Chang 9(6):458–462. 10.1038/s41558-019-0463-3

[26] Grothmann T (2017) Was motiviert zur Eigenvorsorge?: Motivationseffekte von Beteiligungsprozessen in der Klimawandelanpassung. https://www.umweltbundesamt.de/publikationen/was-motiviert-zur-eigenvorsorge. Zugegriffen: 22. Juli 2024

[27] Nationale Präventionskonferenz (2019) Präventionsbericht: nach § 20d Abs. 4 SGB V. https://www.nationale-praeventionskonferenz.de/praeventionsbericht-2019. Zugegriffen: 22. Juli 2024

[28] GKV-Spitzenverband (2022) Leitfaden Prävention. https://gkv-spitzenverband.de/krankenversicherung/praevention_selbsthilfe_beratung/praevention_und_bgf/leitfaden_praevention/leitfaden_praevention.jsp. Zugegriffen: 22. Juli 2024

[29] Lancet Commission EAT (2019) Healthy diets from sustainable food systems: food, planet, health. Summary report of the EAT-Lancet commission. https://eatforum.org/content/uploads/2019/07/EAT-Lancet_Commission_Summary_Report.pdf. Zugegriffen: 22. Juli 2024

[30] Landrigan PJ, Fuller R, Fisher S et al (2019) Pollution and children’s health. Sci Total Environ 650:2389–2394. 10.1016/j.scitotenv.2018.09.375 30292994 10.1016/j.scitotenv.2018.09.375

[31] Xu H, Wen LM, Rissel C (2013) The relationships between active transport to work or school and cardiovascular health or body weight: a systematic review. Asia Pac J Public Health 25(4):298–315. 10.1177/1010539513482488 23572375 10.1177/1010539513482965

[32] Sachverständigenrat für Umweltfragen (2018) Umsteuern erforderlich: Klimaschutz im Verkehrssektor. https://www.umweltrat.de/SharedDocs/Downloads/DE/02_Sondergutachten/2016_2020/2017_11_SG_Klimaschutz_im_Verkehrssektor.html. Zugegriffen: 24. Juli 2024

[33] Sachverständigenrat für Umweltfragen (2020) Für eine aktive und umweltfreundliche Stadtmobilität: Wandel ermöglichen. https://www.umweltrat.de/SharedDocs/Downloads/DE/02_Sondergutachten/2020_Sondergutachten_Stadtmobilitaet/2020_Sondergutachten_Stadtmobilitaet.pdf. Zugegriffen: 22. Juli 2024

[34] Nidens et al. (2024) Kommunikationsleitfaden Kinder vor Hitze schützen. https://hitzeservice.de/wp-content/uploads/2024/05/BMG_Hitze_Leitfaden_Kinder.pdf. Zugegriffen: 24.07.2024

